# Empowering the data science scientist

**DOI:** 10.1186/s13040-021-00246-x

**Published:** 2021-01-23

**Authors:** Jason H. Moore

**Affiliations:** Department of Biostatistics, Epidemiology and Informatics, Perelman School of Medicine, University of Pennsylvania, Philadelphia, PA 19104-6116 USA

The discipline of data science has emerged, flourished, and evolved rapidly over the last 20 years in lockstep with the rise of big data, artificial intelligence, machine learning, statistics, and inexpensive computing. At its core, data science is about integrating the right methods, tools, and technology from different disciplines for the sole purpose of solving a complex data-driven problem in a particular domain such as economics, engineering, or medicine. All data science challenges start with a question. What is the best investment strategy? When will this bridge need to be replaced? Why do some people have adverse reactions to a drug? A key question is “where do these questions come from?”

Most questions arise from domain experts. This is intuitive given economists, engineers, and clinicians have deep knowledge of their specific areas. They know the scientific literature and know where the gaps are. Unfortunately, the trend across disciplines has been to specialize. This, coupled with the rapid expansion of the size of the scientific literature, means that experts are increasingly unaware of key literature outside their specific area. For example, a mechanical engineer working on nanotechnology might be unaware of the mechanical engineering literature in biotechnology. Similarly, a clinician specializing in gastroenterology is not likely keeping up with the latest developments in neurology. The impact of this specialization is that the questions being asked are not informed by literature in other areas.

As the ones who ask the questions, domain experts are usually the scientists leading the research studies. This of course makes sense. An important challenge comes from how data scientists are engaged. Unfortunately, domain experts sometimes see data scientists as service personnel. That is, the data scientist is brought to the project to perform the data management and analysis and then released. There are several issues with this approach. Most obvious is the importance of engaging data scientists early in the development of the research project so that the design of the study is consistent with the analytical approaches to be used. As the great statistician Sir Ronald A. Fisher once said, “To consult the statistician after an experiment is finished is often merely to ask him to conduct a postmortem examination. He can perhaps say what the experiment died of.”

The view of data scientists as service personnel is fading as more and more scientific studies rely on big data for answering questions. It is fair to say that data scientists are increasingly being seen as true collaborators involved in the study design, execution, data analysis, and results interpretation and inference. But what about the formulation of the scientific question itself? This is still most commonly conducted by domain experts. What would scientific studies look like if data scientists asked the questions?

There are several good reasons to believe that data scientists might be in the best position to ask and answer scientific questions. First, data scientists are typically agnostic to application domain. A good data scientist can move fluidly between nanotechnology and biotechnology or gastroenterology and neurology because many of the data challenges are the same. This makes them less biased than a domain expert and perhaps more open to questions others might discount or not think of. Second, data scientists are experts at data and knowledge integration and analysis. This means a data scientist can look across disciplines by integrating data and knowledge from disparate sources including journal publications from different areas. This integration, and any resulting synthesis, could provide the raw materials for asking questions which disciplinarians might not be able to. Finally, being able to see and understand the downstream analytics pipeline could help the data scientist articulate questions which can be answered thus improving the likelihood of success.

It is time to empower the data science scientist with the resources and latitude to articulate important questions and to lead multidisciplinary teams to answer those questions. Successful empowerment must include several key factors. First, domain experts need to recognize the potential for the complementary approach and accept data scientists as leaders of scientific studies. Second, we must provide federal and private funding opportunities for data scientists to be able to collect, integrate, and synthesize the data and knowledge sources from across domains. Here the goal is to generate new questions which is a culture shift from funding designed to execute studies. Questions are the currency of science and therefore must become an important focus of funded activities. Finally, we need to provide the recognition and rewards to encourage data scientists to take on data science scientist roles. Only then can we realize this vision of a no-boundary approach to formulating, asking, and answering the scientific questions which have the biggest impact on society.

## Data Availability

Not applicable.

